# Synergistic Effects in CNTs-PdAu/Pt Trimetallic Nanoparticles with High Electrocatalytic Activity and Stability

**DOI:** 10.1007/s40820-017-0149-1

**Published:** 2017-07-18

**Authors:** Xin-Lei Cai, Chang-Hai Liu, Jie Liu, Ying Lu, Ya-Nan Zhong, Kai-Qi Nie, Jian-Long Xu, Xu Gao, Xu-Hui Sun, Sui-Dong Wang

**Affiliations:** 1 Institute of Functional Nano and Soft Materials (FUNSOM), Jiangsu Key Laboratory for Carbon-Based Functional Materials and Devices, Soochow University, Suzhou, 215123 Jiangsu People’s Republic of China; 2 School of Materials Science and Engineering, Jiangsu Collaborative Innovation Center of Photovoltaic Science and Engineering, Changzhou University, Changzhou, 213164 Jiangsu People’s Republic of China

**Keywords:** CNTs, PdAu/Pt, Trimetallic nanoparticles, Methanol oxidation reaction, Electrocatalytic activity, Synergistic effects

## Abstract

We present a straightforward physical approach for synthesizing multiwalled carbon nanotubes (CNTs)-PdAu/Pt trimetallic nanoparticles (NPs), which allows predesign and control of the metal compositional ratio by simply adjusting the sputtering targets and conditions. The small-sized CNTs-PdAu/Pt NPs (~3 nm, Pd/Au/Pt ratio of 3:1:2) act as nanocatalysts for the methanol oxidation reaction (MOR), showing excellent performance with electrocatalytic peak current of 4.4 A mg_Pt_^−1^ and high stability over 7000 s. The electrocatalytic activity and stability of the PdAu/Pt trimetallic NPs are much superior to those of the corresponding Pd/Pt and Au/Pt bimetallic NPs, as well as a commercial Pt/C catalyst. Systematic investigation of the microscopic, crystalline, and electronic structure of the PdAu/Pt NPs reveals alloying and charge redistribution in the PdAu/Pt NPs, which are responsible for the promotion of the electrocatalytic performance.

**Electronic supplementary material:**

The online version of this article (doi:10.1007/s40820-017-0149-1) contains supplementary material, which is available to authorized users.

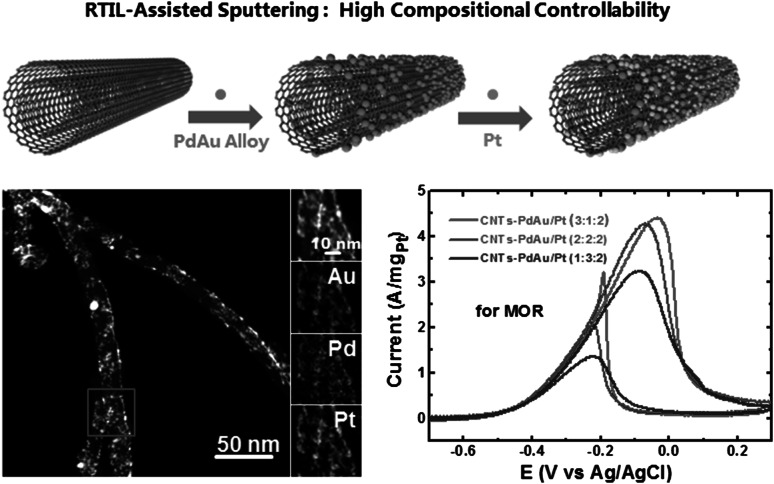

## Highlights

- CNTs-PdAu/Pt trimetallic nanoparticles (NPs, ~3 nm) were synthesized using a straightforward physical approach of RTILs-assisted sputtering deposition.
- As a high-performance nanocatalyst for the methanol oxidation reaction (MOR), CNTs-PdAu/Pt NPs show an electrocatalytic peak current of up to 4.4 A mg_Pt_^−1^ and high stability over 7000 s, which is much superior to those of Pt-based bimetallic NPs and a commercial Pt/C catalyst. The optimal atomic ratio of Pd/Au/Pt, which has the best catalytic performance, was found to be 3:1:2.
- Synergistic effects arose from charge redistribution among Pd, Au, and Pt in CNTs-PdAu/Pt NPs may be responsible for the promotion of the electrocatalytic activity.

## Introduction

Target-oriented design and controlled synthesis of noble-metal nanoparticles (NPs) have aroused extensive attention because of important applications in diverse fields, such as nanocatalysis [1–3], chemical sensing [4, 5], and drug delivery [6, 7]. In particular, Pt-based NPs are still the most efficient catalytic materials in clean-energy technologies such as fuel cells [8–10]. For minimizing Pt consumption and optimizing catalytic performance of Pt-based NPs, tremendous efforts have been devoted to synthesize Pt-based multi-metallic NPs because of their superior selectivity, activity, and/or stability in comparison with their monometallic counterparts [11–20].

Synergistic effects of Pt-based bimetallic NPs (such as AuPt [15–17] and PdPt [18–20] NPs) have been well documented. However, Pt-based trimetallic NPs have not been sufficiently explored, as the presence of multiple components increases the complexity of the controlled NP synthesis and thorough characterization. In-depth probing into Pt-based trimetallic NPs could provide new insights into the correlation between the composition, structure, and catalytic properties of noble-metal nanocatalysts.

Kotaro et al. reported an electrocatalyst comprising Pt monolayers on PdAu alloy NPs, which exhibited highly durable and active catalytic performance toward the oxygen reduction reaction (ORR) [21]. Shin et al. synthesized Au@PdPt core–shell NPs and observed better catalytic activity than bimetallic core–shell NPs toward the methanol oxidation reaction (MOR) [22]. Zhang et al. proposed a theoretical model in which the catalytic activity of alloy-core@Pt NPs varies linearly with the alloy–core composition [23]. Nevertheless, two great challenges remain in the experimental study of Pt-based trimetallic NPs. One is precise control of the compositional ratio of metals by chemical approaches that involve the different reduction kinetics of metallic precursors. The other is elucidation of the dominant synergistic effects in the complex ternary nanostructures [24, 25].

Room temperature ionic liquids (RTILs)-assisted sputtering is a straightforward physical approach to prepare monometallic and bimetallic NPs in an environmental-friendly and by-product-free manner [26, 27]. Various bimetallic NPs with different composition can be synthesized by varying the composition of metal targets without any chemical additives (such as NaBH_4_ and citric acid). For example, Au@Ag and Pd@Ag core–shell NPs, PtNi and AuPd alloy NPs have been successfully prepared by RTILs-assisted sputtering on various nanosupports, such as graphene, carbon nanotubes (CNTs), and TiO_2_ NPs [14, 28–30]. However, it is more challenging to prepare trimetallic NPs using sputtering due to the increased difficulty in controlling their morphology and composition.

In this report, we prepared uniform PdAu/Pt trimetallic NPs decorated on CNTs using a RTILs-assisted sputtering method. CNTs are herein used as the nanosupport, since it has been reported that the high conductivity and huge surface area of CNTs are beneficial for electron transfer and mass transport involved in the MOR [10, 31, 32]. The composition and the catalytic behavior of CNTs-PdAu/Pt NPs were controlled by simply varying the sputtering conditions. The electrocatalytic activity and stability of CNTs-PdAu/Pt NPs toward the MOR were systematically investigated and compared with corresponding Pd/Pt and Au/Pt bimetallic NPs and a commercial Pt/C catalyst. Synergistic effects in the CNTs-PdAu/Pt NPs were also discussed.

## Experimental

### Chemicals and Materials

All chemicals were analytical. Commercial Pt/C (20 wt%) catalyst, KOH, and methanol were purchased from Alfa Aesar and used as received. The RTIL, 1-butyl-3-methylimidazolium tetrafluoroborate ([BMIm][BF_4_], purity > 99%), was purchased from Shanghai Cheng Jie Chemical and purified under vacuum for 24 h before use. CNTs with diameters of 30–50 nm were purchased from Nanjing XFNANO Materials Tech.

### Preparation of CNTs-Supported NPs

**Scheme 1:**
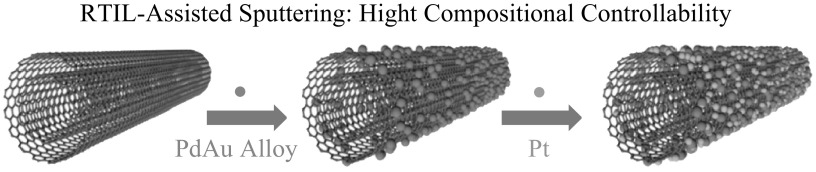
Process used to prepare CNTs-PdAu/Pt NPs

All the metal NPs were synthesized by a RTILs-assisted sputtering approach and then self-decorated on CNTs in RTILs [14, 28–30]. Firstly, 10 mg of CNTs were completely dispersed in 2 mL [BMIm][BF_4_] to form a CNT-RTIL suspension in a clean stainless-steel pot. Then, selected metals were sputtered onto the CNT-RTIL suspension using a desktop direct-current sputtering system (Quorum Technologies, equipped with a quartz microbalance thickness monitor). For all the metals, the metal deposition rate was kept at about 0.2 Å s^−1^ and the working pressure was 0.01 mbar. In order to make a systematic comparison, monometallic, bimetallic, and trimetallic NPs of interest were prepared. For monometallic samples, Au, Pd, and Pt targets were sputtered for 15 min to synthesize the Au, Pd, and Pt NPs, respectively. For bimetallic samples, the Au/Pt NPs were synthesized by successively sputtering Au for 10 min and Pt for 5 min; the Pd/Pt NPs were synthesized by successively sputtering Pd for 10 min and Pt for 5 min; the PdAu NPs were synthesized by sputtering a PdAu alloy target (with Pd/Au ratio of 1:1) for 15 min. As for trimetallic samples, as illustrated in Scheme 1, the PdAu/Pt NPs were synthesized by successively sputtering PdAu alloy and Pt onto the CNT-RTIL suspension. A PdAu alloy target, with a Pd/Au ratio of 1:3, 1:1, or 3:1, was sputtered for 10 min. Afterwards, Pt was sputtered for 5 min onto the suspension containing PdAu. The present design is to obtain different compositional ratios of Pd/Au/Pt, which is utilized to study the synergistic effects of multiple metal components. Finally, all the CNTs-supported NPs were extracted from [BMIm][BF_4_] using high-speed centrifugation and decantation, followed by multiple washing in acetone aided by bath ultrasonication. The final products were dry black powder and used as the nanocatalysts.

### Characterization Techniques

The compositional ratio of the metal components was measured using inductively coupled plasma atom emission spectroscopy (ICP-AES, Vista-MPX). The microscopic structure of the CNTs-supported NPs was characterized using high-resolution transmission electron microscopy (HRTEM, FEI Tecnai G2) and high-angle annular dark-field scanning TEM (HAADF-STEM). The crystalline structure of the CNTs and the CNTs-supported NPs was analyzed by X-ray diffraction (XRD, PANalytical Empyrean) with Cu *Kα* radiation (*λ* = 1.5418 Å). The electronic structure of the CNTs-supported NPs was probed by X-ray photoelectron spectroscopy (XPS, Kratos Axis Ultra DLD) under ultra-high vacuum and X-ray absorption near-edge spectroscopy (XANES) at the Taiwan Light Source (TLS).

### Electrochemical Measurements

Cyclic voltammetry (CV) and chronoamperometry (CA) measurements were taken using a CHI660E electrochemical workstation with glassy carbon (GC), Ag/AgCl, and a Pt wire as the working, reference, and counter electrodes, respectively. Prior to being coated by the nanocatalysts, the GC electrode was polished using alumina slurry, washed ultrasonically in ethanol and water, and then dried at room temperature (27 °C). The as-prepared nanocatalysts (1 mg) were dispersed in a mixture of 500 μL ethanol and 5 μL Nafion solution (5 wt%) under ultrasonication for 30 min. Afterwards, 10 μL of the suspension was coated onto the GC electrode surface. Prior to electrochemical measurements, the nanocatalysts were activated in 1 M KOH to remove any dissolved oxygen and release active sites. CV tests for the MOR were performed between –0.7 and 0.3 V (vs. Ag/AgCl) at room temperature (27 °C) in an electrolyte containing 1 M KOH and 1 M CH_3_OH.

## Results and Discussion

### Morphology, Composition, and Structure of CNTs-PdAu/Pt NPs

The composition and content of CNTs-PdAu/Pt NPs can be controlled by adjusting the sputtering conditions. The elemental contents of Pd, Au, and Pt determined by ICP-AES are listed in Table 1. One can see that the measured compositional ratios, for both the trimetallic NPs and the compared bimetallic NPs, closely match the experimentally designed ones.

**Table 1:** Elemental content of Pd, Au, and Pt in CNTs-supported nanocatalysts as determined by ICP-AES measurements

CNTs-supported nanocatalyst	Pd (mg L^−1^)	Au (mg L^−1^)	Pt (mg L^−1^)	Atomic ratio
PdAu/Pt (3:1:2)	4.36	3.15	5.82	2.95:1.07:2
PdAu/Pt (2:2:2)	2.98	5.49	5.59	1.95:1.95:2
PdAu/Pt (1:3:2)	1.55	9.77	5.61	1.01:3.45:2
Pd/Pt	4.20	0	3.86	2.00:1
Au/Pt	0	11.62	4.40	2.62:1

**Fig. 1:**
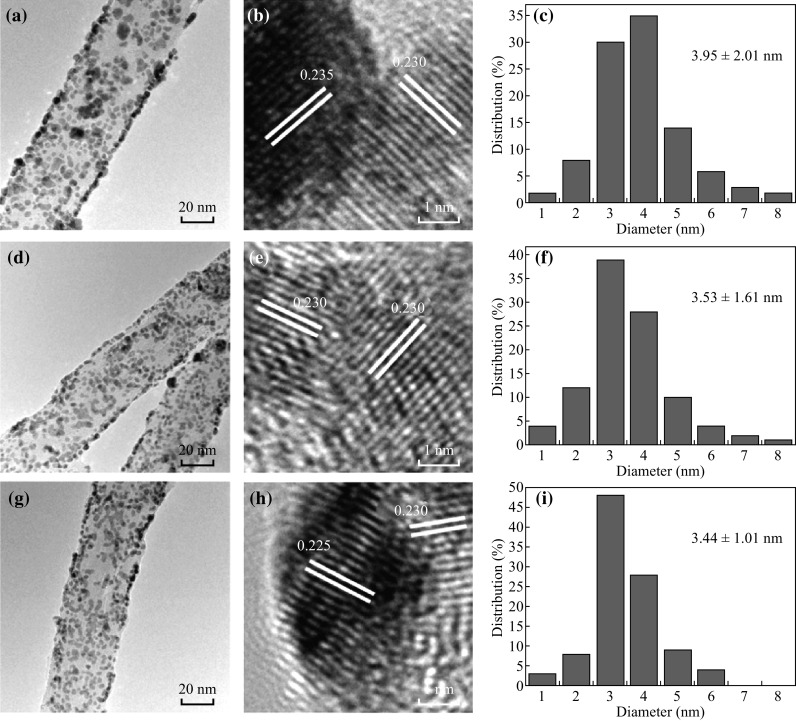
TEM and HRTEM images of CNTs-PdAu/Pt trimetallic NPs and their size distribution with different Pd/Au/Pt ratios: **a**, **b, c** 1:3:2, **d**, **e, f** 2:2:2, and **g**, **h, i** 3:1:2

Figure 1 shows the TEM and HRTEM images of the CNTs-PdAu/Pt NPs with different Pd/Au/Pt ratios as well as the size distribution of NPs. Regardless of Pd/Au/Pt ratio, all of the PdAu/Pt NPs show a similar interplanar spacing of about 0.23 nm, which matches the (111) planes of a face-centered cubic (*fcc*) lattice of no matter Au, Pd, or Pt [13, 18]. The mean size and variance of the NPs were calculated from measured data of ~400 randomly picked NPs in the TEM images for each sample. It can be seen that the NPs are decorated and dispersed uniformly on the nanotubes, and the size of NPs varies with the ratio of Pd/Au/Pt. For PdAu/Pt NPs with Pd/Au/Pt ratio of 1:3:2 [denoted as PdAu/Pt (1:3:2)], the mean size is 3.95 nm, whereas, for PdAu/Pt (2:2:2) and PdAu/Pt (3:1:2) NPs, the mean size is, respectively, 3.53 and 3.44 nm. The size dependence on Pd/Au/Pt ratio may be related to the interaction between the different metals and the CNTs [27, 29], as a strong interaction would prevent small-sized NPs from aggregation and coalescence. The decreased size with increased Pd content in Fig. 1 may result from a stronger interaction between Pd and the CNTs, compared with that between Au and the CNTs. Our previous works demonstrated that CNTs-Pd monometallic NPs are typically smaller than CNTs-Au NPs prepared by the present sputtering approach [27–29]. In addition, CNTs-Pd/Pt bimetallic NPs also possess smaller size compared with CNTs-Au/Pt NPs, as demonstrated in Fig. S1.

**Fig. 2:**
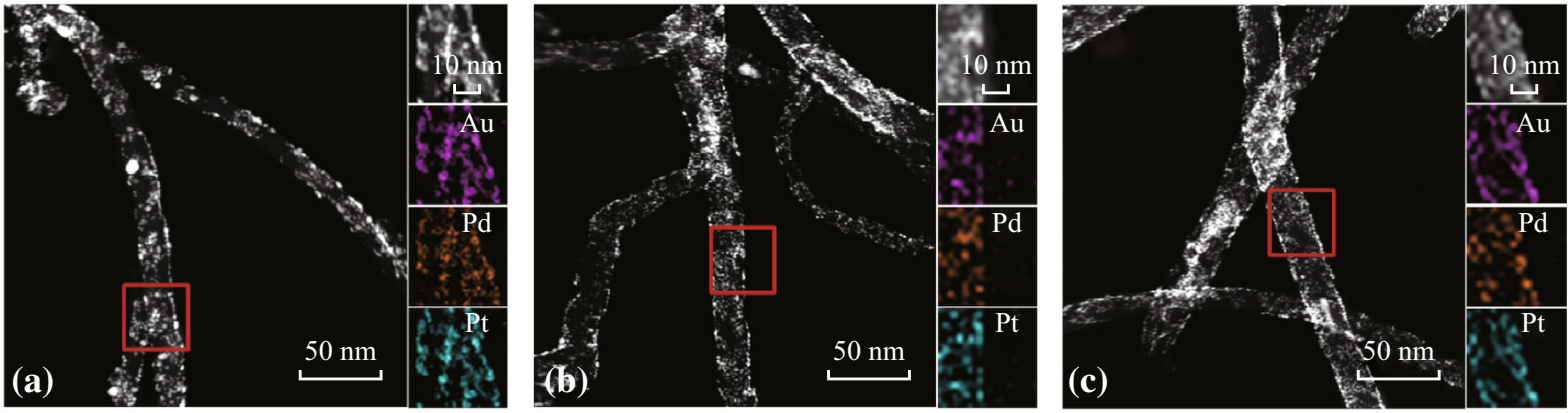
HAADF-STEM images and regional elemental mapping of the Au, Pd, and Pt content of CNTs-PdAu/Pt trimetallic NPs with different Pd/Au/Pt ratios: **a** 1:3:2, **b** 2:2:2, and **c** 3:1:2

The elemental spatial distributions of Au, Pd, and Pt on the CNTs are shown in Fig. 2. Au, Pd, and Pt are uniformly dispersed on the CNTs, suggesting atomic intermixing of Pd, Au, and Pt despite of two-step sputtering [33–35]. Previous reports demonstrated that the successive RTILs-assisted sputtering of two noble metals formed bimetallic alloyed NPs [36, 37].

**Fig. 3:**
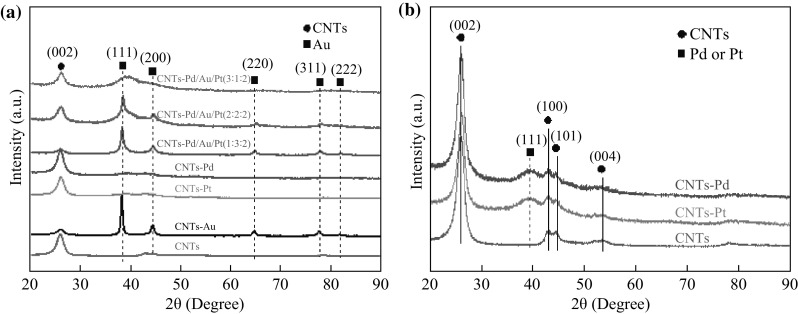
XRD patterns of **a** CNTs-PdAu/Pt trimetallic NPs with different Pd/Au/Pt ratios and the CNTs-supported Au, Pd, and Pt monometallic NPs and pristine CNTs. **b** A magnified view of CNTs-supported Pd and Pt monometallic NPs and pristine MWCNTs shown in **a**

The XRD patterns of CNTs trimetallic, bimetallic, monometallic NPs, and pristine CNTs are shown in Fig. 3a and Fig. S2 for comparison. The diffraction peak at 26.2° is corresponding to the (002) plane of CNTs [10, 29], and the NP decoration does not affect the crystallinity of the CNTs. The diffraction peaks of CNTs-Au NPs at 38.2°, 44.4°, 64.6°, 77.8°, and 81.8° can be assigned to Au (111), (200), (220), (311), and (222), respectively [14, 17]. However, the peaks of CNTs-Pd and CNTs-Pt NPs are rather weak and have a broad full-width at half-maximum (FWHM), as shown in Fig. 3b which is a magnified view of the pristine CNTs, CNTs-Pd, and CNTs-Pt samples in Fig. 3a. This may be due to lower crystallinity of the Pd and Pt ensembles arising from their small size and partial surface oxidation [32, 37]. Significantly, upon increasing the Pd content in CNTs-PdAu/Pt NPs, the Au (111) peak gradually broadens and slightly shifts toward larger 2*θ* (Fig. 3a), implying alloy formation to a large extent in the PdAu/Pt NPs [37, 38].

**Fig. 4:**
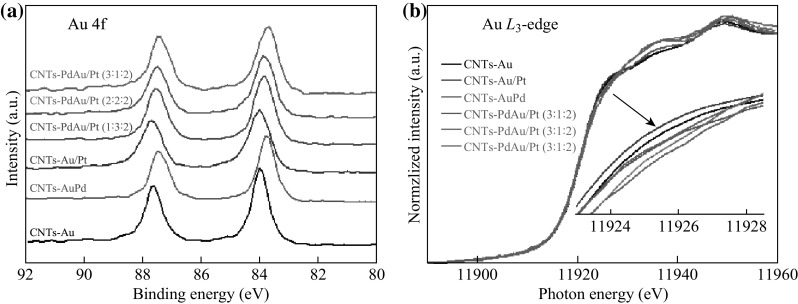
**a** Au 4*f* XPS spectra and **b** normalized Au *L*_3_-edge XANES spectra of CNTs-PdAu/Pt trimetallic NPs with different Pd/Au/Pt ratios, Au/Pt bimetallic NPs, and Au NPs. The *insets* in **b** are magnified white line regions of as-obtained samples

For an in-depth understanding of the synergistic effects in CNTs-PdAu/Pt NPs, the electronic structure of CNTs trimetallic, bimetallic, and monometallic NPs was investigated by XPS and XANES. Figure 4a presents the Au 4*f* XPS spectra of the samples. It can be noticed that the Au 4*f* peaks shift slightly toward higher binding energy in CNTs-Au/Pt NPs compared with CNTs-Au NPs, which is a result of electron transfer from Au to Pt [39–41]. The opposite situation was observed in CNTs-PdAu-alloyed NPs with a negative shift of the Au 4*f* peaks, as Au gains electrons from Pd [13, 42]. In the case of CNTs-PdAu/Pt NPs, increasing the Pd content results in a gradual shift of the Au 4*f* peaks toward lower binding energy. As evident from Fig. 4a, the Au 4*f* peaks of PdAu/Pt (2:2:2) NPs are located between those of CNTs-Au NPs and CNTs-PdAu NPs, indicating the concurrence of Au–Pd and Au–Pt interactions in the CNTs-PdAu/Pt NPs.

To further probe the electronic structure of Au, Fig. 4b shows the Au *L*_3_-edge XANES spectra of the samples, with an inset of a magnified view of the white line region around 11,925 eV (2*p*-to-5*d* transition) [28, 37, 40, 41]. The normalized white line intensity reflects the density of Au *d*-band holes, and a higher intensity corresponds to more *d*-band holes [42]. The XANES spectra indicate that Au–Pt interactions increase the Au white line intensity (Au *d*-band holes increase due to electron transfer from Au to Pt), while Au–Pd interactions do the contrary (Au *d*-hole depletion due to electron transfer from Pd to Au). For the CNTs-supported trimetallic NPs, the Au white line intensity decreases with increase in the Pd content. The PdAu/Pt (3:1:2) NPs show the lowest white line intensity, which results from that, at this Pd/Au/Pt ratio, Au is surrounded by Pd and thus gains a number of electrons from Pd. The trend revealed by the XANES results is well consistent with the XPS analysis: electron transfer occurs from Au to Pt and from Pd to Au, and this electron redistribution is dependent on compositional ratio of the CNTs-PdAu/Pt NPs.

**Fig. 5:**
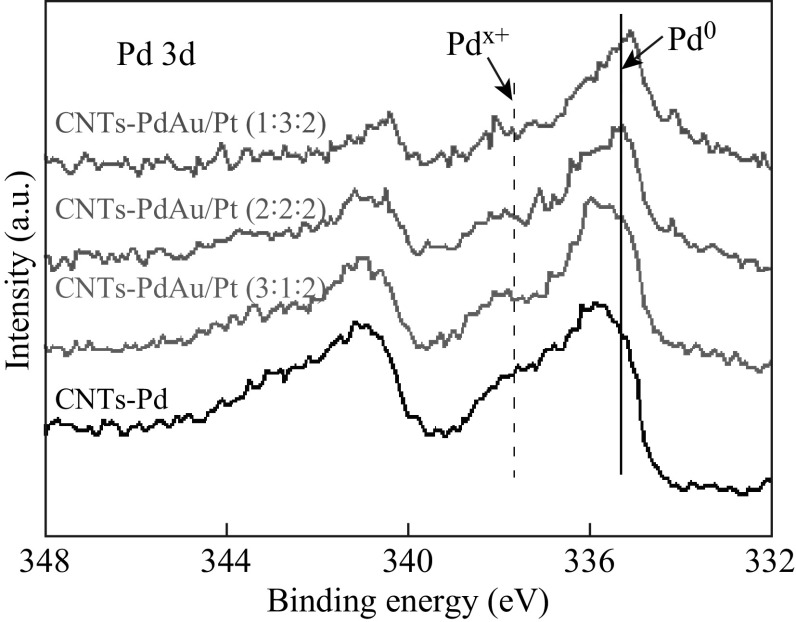
Pd 3*d* XPS spectra of CNTs-supported PdAu/Pt trimetallic NPs with different Pd/Au/Pt ratios and Pd NPs

On the other hand, Pd 3*d* XPS spectra for the same CNTs-PdAu/Pt NPs are illustrated in Fig. 5, where the corresponding spectrum of CNTs-Pd NPs is shown as a reference. As Pd may be partially oxidized, both metallic Pd^0^ and oxidized Pd^x+^ features were observed in the XPS spectra. Using the Pd 3*d*_5/2_ peak as an example, the Pd^0^ and Pd^x+^ components are at binding energies of about 335.5 and 337.5 eV (Fig. 5), respectively [35, 43]. With reducing the Pd content, the relative intensity of the Pd^x+^ component gradually decreases and the Pd^0^ one becomes dominant; this can be ascribed to the significantly improved oxidation resistance of Pd upon alloying with Au [14, 28].

**Fig. 6:**
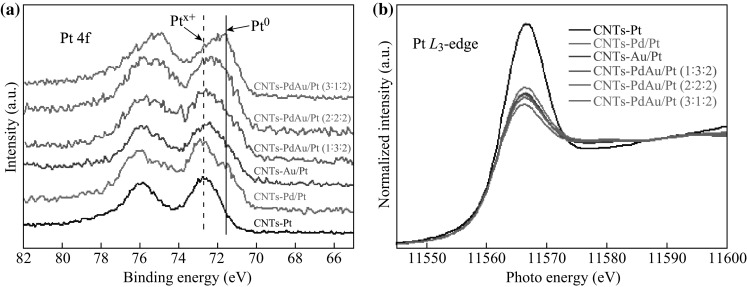
**a** Pt 4*f* XPS spectra and **b** Pt *L*_3_-edge XANES spectra of CNTs-supported PdAu/Pt trimetallic NPs with different Pd/Au/Pt ratios, Au/Pt and Pd/Pt bimetallic NPs, and Pt NPs

Significantly, a similar evolution is present in the Pt 4*f*_7/2_ XPS spectra (Fig. 6a) for CNTs trimetallic, bimetallic, and monometallic NPs, where the binding energies of metallic Pt^0^ and oxidized Pt^x+^ features are about 71.6 and 72.8 eV, respectively [29, 43], whereas, for CNTs-Pt monometallic NPs, the Pt 4*f* peaks have a large FWHM and the Pt^x+^ component appears to dominate the spectrum. This is presumably due to the small size effect and partial surface oxidation of the CNTs-Pt NPs [32, 44]. With adding Pd and/or Au, the Pt^0^ component emerges and gradually becomes dominant upon further increasing the Pd content. This is in good agreement with the electronic structure results for Au and Pd, indicating that Pt gains electrons and has enhanced stability against surface oxidation in the CNTs-PdAu/Pt NPs. This hypothesis is verified by the Pt *L*_3_-edge XANES spectra (Fig. 6b), in which the white line related to the 2*p*-to-5*d* transition is located around 11,566 eV [3, 41]. The white line intensity decreases dramatically with adding Pd and/or Au, which can be a result of electron transfer from Pd and/or Au to Pt. Furthermore, the extent of electron loss is larger for Pd than that for Au, as the trimetallic NPs with the highest Pd content [PdAu/Pt (3:1:2)] show the lowest white line intensity. Based on these results, we conclude that significant charge redistribution occurs in CNTs-PdAu/Pt NPs due to alloying of the metals, which is expected to have a great influence on their catalytic properties.

### Catalytic Performance of CNTs-PdAu/Pt Trimetallic NPs

**Fig. 7:**
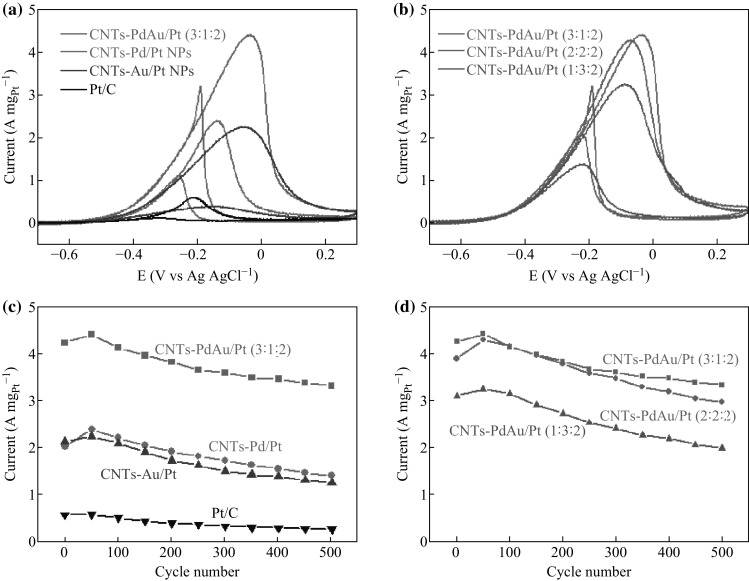
CV curves for the MOR using: **a** CNTs-PdAu/Pt (3:1:2) NPs, Pd/Pt and Au/Pt bimetallic NPs, and a commercial Pt/C catalyst, and **b** CNTs-PdAu/Pt trimetallic NPs with different Pd/Au/Pt ratios. The durability (500 cycles) of the oxidation peak current for: **c** samples shown in **a**; and **d** samples shown in **b**

The catalytic performance of CNTs-supported trimetallic, bimetallic, and monometallic NPs for the MOR was evaluated under alkaline conditions, and a commercial Pt/C catalyst was used as a reference. As shown in Fig. 7a, b, Pt-mass-normalized CV curves exhibit the oxidation peaks in both forward and reverse scans. The oxidation peak current density in forward scan (*I*_f_) gives an indication of electrocatalytic activity toward the MOR, with a larger *I*_f_ value corresponding to higher activity [32, 35]. Firstly, *I*_f_ for the PdAu/Pt (3:1:2) trimetallic NPs (4.4 A mg_Pt_^−1^) is much larger than those for the corresponding Pd/Pt (2.4 A mg_Pt_^−1^) and Au/Pt (2.2 A mg_Pt_^−1^) bimetallic NPs. The CV results demonstrate the far superior electrocatalytic activity of the trimetallic NPs. It should be noted that the CNTs-supported trimetallic and bimetallic NPs are highly active, since *I*_f_ for the commercial Pt/C catalyst is only 0.6 A mg_Pt_^−1^. Secondly, *I*_f_ for the CNTs-PdAu/Pt NPs is dependent on the Pd/Au/Pt ratio. With increasing the Pd content, *I*_f_ increases from 3.2 to 4.2 then to 4.4 A mg_Pt_^−1^ for the PdAu/Pt (1:3:2), (2:2:2), and (3:1:2) NPs, respectively. The Pt-mass-normalized *I*_f_ value of 4.4 A mg_Pt_^−1^ is among the best performance reported for the MOR nanocatalysts [10, 20, 32]. Therefore, precise control of the compositional ratio is crucial for catalytic applications of these trimetallic NPs (a volcano plot of *I*_f_ for the nanocatalysts with different compositional ratio is shown in Fig. S3).

**Fig. 8:**
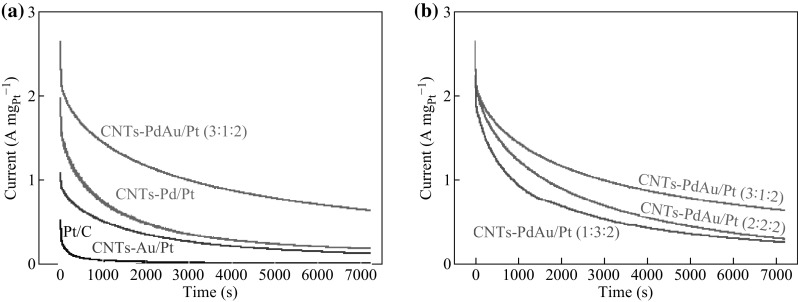
CA curves for the MOR (–0.2 V vs. Ag/AgCl in 1 M CH_3_OH + 1 M KOH) using: **a** CNTs-PdAu/Pt (3:1:2) NPs, Pd/Pt, and Au/Pt bimetallic NPs, and a commercial Pt/C catalyst, and **b** MWCNTs-supported PdAu/Pt trimetallic NPs with different Pd/Au/Pt ratios

The durability measurements for the nanocatalysts are presented in Fig. 7c, d; all of which show a similar trend in *I*_f_ change. Regarding the trimetallic NPs, *I*_f_ is relatively stable up to 500 CV cycles, with 76%, 69%, and 61% retention for the PdAu/Pt (3:1:2), (2:2:2), and (1:3:2) NPs, respectively. However, *I*_f_ is reduced to 59%, 56%, and 47% of the initial values for the corresponding Pd/Pt and Au/Pt bimetallic NPs, and a commercial Pt/C catalyst, respectively. Moreover, Fig. 8a, b shows Pt-mass-normalized CA curves at –0.2 V versus Ag/AgCl for the MOR. The CNTs-supported trimetallic NPs exhibit superior catalytic stability over 7000 s compared with their bimetallic counterparts and a commercial Pt/C catalyst, and the trimetallic NPs with the highest Pd content [PdAu/Pt (3:1:2)] show the greatest catalytic stability.

By combining the comprehensive characterization and catalytic testing results, we can understand the synergistic effects in the CNTs-PdAu/Pt NPs. On the one hand, alloying in the CNTs-PdAu/Pt NPs may create a number of tiny Pt ensembles, whose surfaces will act as catalytically active sites for the MOR [13]. On the other hand, adding Au stabilizes Pt against surface oxidation, and adding Pd induces significant electron transfer from Pd to Pt. The latter effect is critical to the modification of electronic structure of Pt, where gaining electrons from Pd is expected to cause a downshift in Pt *d*-band center relative to the Fermi level [22, 45]. As a consequence, the reduced density of empty states in Pt *d*-band will weaken the interaction between Pt and the MOR intermediates (such as CO), thus suppressing the CO poisoning of the trimetallic nanocatalysts [13, 28]. This combination of the effects explains well the observation that the CNTs-supported PdAu/Pt (3:1:2) NPs, with the highest Pd content (corresponding to the most electron gain for Pt), possess the highest catalytic activity and stability toward the MOR.

## Conclusion

A successive RTILs-assisted sputtering technique was utilized to synthesize CNTs-supported PdAu/Pt trimetallic NPs with a small size and tunable composition. With an optimal Pd/Au/Pt ratio of 3:1:2, the PdAu/Pt NPs achieve an electrocatalytic peak current of up to 4.4 A mg_Pt_^−1^ and high stability over 7000 s toward the MOR, which is much superior to those of bimetallic control samples and a commercial Pt/C catalyst. The excellent electrocatalytic performance of this ternary nanocatalyst is ascribed to the synergistic effects arising from favorable charge redistribution among the Pd, Au, and Pt ensembles. Adding Au improves the stability of the catalytically active Pt surface, and adding Pd enhances its resistance to the CO poisoning. The approach presented here offers a simple strategy to predesign and tailor the composition of CNTs-supported trimetallic NPs for catalysis and energy applications.

## Electronic supplementary material

Below is the link to the electronic supplementary material.

Supplementary material 1 (PDF 689 kb)

## References

[1] Liu P, Zhao Y, Qin R, Mo S, Chen G (2016). Photochemical route for synthesizing atomically dispersed palladium catalysts. Science.

[2] Zhang Y, Cui X, Shi F, Deng Y (2011). Nano-gold catalysis in fine chemical synthesis. Chem. Rev..

[3] Cheng N, Stambula S, Wang D, Banis MN, Liu J (2016). Platinum single-atom and cluster catalysis of the hydrogen evolution reaction. Nat. Commun..

[4] He L, Liu Y, Liu J, Xiong Y, Zheng J, Liu Y, Tang Z (2013). Core–shell noble-metal@metal-organic-framework nanoparticles with highly selective sensing property. Angew. Chem. Int. Ed..

[5] Zheng T, Bott S, Huo Q (2016). Techniques for accurate sizing of gold nanoparticles using dynamic light scattering with particular application to chemical and biological sensing based on aggregate formation. ACS Appl. Mater. Interfaces.

[6] Arvizo RR, Bhattacharyya S, Kudgus RA, Giri K, Bhattacharya R, Mukherjee P (2012). Intrinsic therapeutic applications of noble metal nanoparticles: past, present and future. Chem. Soc. Rev..

[7] Wang F, Wang Y-C, Dou S, Xiong M-H, Sun T-M, Wang J (2011). Doxorubicin-tethered responsive gold nanoparticles facilitate intracellular drug delivery for overcoming multidrug resistance in cancer cells. ACS Nano.

[8] Zeng Z, Tan C, Huang X, Bao S, Zhang H (2014). Growth of noble metal nanoparticles on single-layer TiS_2_ and TaS_2_ nanosheets for hydrogen evolution reaction. Energy Environ. Sci..

[9] Li T, You H-J, Xu M-W, Song X-P, Fang J-X (2012). Electrocatalytic properties of hollow coral-like platinum mesocrystals. ACS Appl. Mater. Interfaces.

[10] Wu B, Hu D, Kuang Y, Liu B, Zhang X, Chen J (2009). Functionalization of carbon nanotubes by an ionic-liquid polymer: dispersion of Pt and PtRu nanoparticles on carbon nanotubes and their electrocatalytic oxidation of methanol. Angew. Chem. Int. Ed..

[11] Kakati N, Maiti J, Lee SH, Jee SH, Viswanathan B, Yoon YS (2014). Anode catalysts for direct methanol fuel cells in acidic media: Do we have any alternative for Pt or Pt–Ru?. Chem. Rev..

[12] You H-J, Fang J-X (2016). Particle-mediated nucleation and growth of solution-synthesized metal nanocrystals: a new story beyond the LaMer curve. Nano Today.

[13] Gao F, Goodman DW (2012). Pd-Au bimetallic catalysts: understanding alloy effects from planar models and (supported) nanoparticles. Chem. Soc. Rev..

[14] Chang J-B, Liu C-H, Liu J, Zhou Y-Y, Gao X, Wang S-D (2015). Green-chemistry compatible approach to TiO_2_-supported PdAu bimetallic nanoparticles for solvent-free 1-phenylethanol oxidation under mild conditions. Nano-Micro Lett..

[15] Luo J, Njoki PN, Lin Y, Mott D, Wang L, Zhong C-J (2006). Characterization of carbon-supported AuPt nanoparticles for electrocatalytic methanol oxidation reaction. Langmuir.

[16] Zhang H, Toshima N (2013). Synthesis of Au/Pt bimetallic nanoparticles with a Pt-rich shell and their high catalytic activities for aerobic glucose oxidation. J. Colloid Interface Sci..

[17] You H-J, Zhang F-L, Liu Z, Fang J-X (2014). Free-standing Pt–Au hollow nanourchins with enhanced activity and stability for catalytic methanol oxidation. ACS Catal..

[18] Zhang H, Jin M, Xia Y (2012). Enhancing the catalytic and electrocatalytic properties of Pt-based catalysts by forming bimetallic nanocrystals with Pd. Chem. Soc. Rev..

[19] You H-J, Wang W-J, Yang S-C (2014). A universal rule for organic ligand exchange. ACS Appl. Mater. Interfaces.

[20] Wang L, Nemoto Y, Yamauchi Y (2011). Direct synthesis of spatially-controlled Pt-on-Pd bimetallic nanodendrites with superior electrocatalytic activity. J. Am. Chem. Soc..

[21] Sasaki K, Naohara H, Choi Y, Cai Y, Chen W-F, Liu P, Adzic RR (2012). Highly stable Pt monolayer on PdAu nanoparticle electrocatalysts for the oxygen reduction reaction. Nat. Commun..

[22] Kang SW, Lee YW, Park Y, Choi B-S, Hong JW, Park K-H, Han SW (2013). One-pot synthesis of trimetallic Au@PdPt core–shell nanoparticles with high catalytic performance. ACS Nano.

[23] Zhang L, Iyyamperumal R, Yancey DF, Crooks RM, Henkelman G (2013). Design of Pt-shell nanoparticles with alloy cores for the oxygen reduction reaction. ACS Nano.

[24] Zhang H-J, Cao Y-N, Lu L-L, Cheng Z, Zhang S-W (2015). Trimetallic Au/Pt/Rh nanoparticles as highly active catalysts for aerobic glucose oxidation. Metall. Mater. Trans. B.

[25] Zhang H-J, Lu L-L, Cao Y-N, Du S, Cheng Z, Zhang S-W (2014). Fabrication of catalytically active Au/Pt/Pd trimetallic nanoparticles by rapid injection of NaBH_4_. Mater. Res. Bull..

[26] Wender H, de Oliveira LF, Migowski P, Feil AF, Lissner E, Prechtl MH, Teixeira SR, Dupont J (2010). Ionic liquid surface composition controls the size of gold nanoparticles prepared by sputtering deposition. J. Phys. Chem. C.

[27] Liu C-H, Mao B-H, Gao J, Zhang S, Gao X, Liu Z, Lee S-T, Sun X-H, Wang S-D (2012). Size-controllable self-assembly of metal nanoparticles on carbon nanostructures in room-temperature ionic liquids by simple sputtering deposition. Carbon.

[28] Liu C-H, Liu R-H, Sun Q-J, Chang J-B, Gao X, Liu Y, Lee S-T, Kang Z-H, Wang S-D (2015). Controlled synthesis and synergistic effects of graphene-supported PdAu bimetallic nanoparticles with tunable catalytic properties. Nanoscale.

[29] Zhou Y-Y, Liu C-H, Liu J, Cai X-L, Lu Y, Zhang H, Sun X-H, Wang S-D (2016). Self-decoration of PtNi alloy nanoparticles on multiwalled carbon nanotubes for highly efficient methanol electro-oxidation. Nano-Micro Lett..

[30] Yoshii K, Tsuda T, Arimura T, Imanishi A, Torimoto T, Kuwabata S (2012). Platinum nanoparticle immobilization onto carbon nanotubes using Pt-sputtered room-temperature ionic liquid. RSC Adv..

[31] Zhao Y, Fan L, Zhong H, Li Y, Yang S (2007). Platinum nanoparticle clusters immobilized on multiwalled carbon nanotubes: electrodeposition and enhanced electrocatalytic activity for methanol oxidation. Adv. Funct. Mater..

[32] Shen Y, Xiao K, Xi J, Qiu X (2015). Comparison study of few-layered graphene supported platinum and platinum alloys for methanol and ethanol electro-oxidation. J. Power Sources.

[33] Wang L, Yamauchi Y (2010). Autoprogrammed synthesis of triple-layered Au@ Pd@Pt core-shell nanoparticles consisting of a Au@Pd bimetallic core and nanoporous Pt shell. J. Am. Chem. Soc..

[34] Wang L, Yamauchi Y (2011). Strategic synthesis of trimetallic Au@ Pd@ Pt core–shell nanoparticles from poly (vinylpyrrolidone)-based aqueous solution toward highly active electrocatalysts. Chem. Mater..

[35] Dutta S, Ray C, Sasmal AK, Negishi Y, Pal T (2016). Fabrication of dog-bone shaped Au NR core–Pt/Pd shell trimetallic nanoparticle-decorated reduced graphene oxide nanosheets for excellent electrocatalysis. J. Mater. Chem. A.

[36] Okazaki K, Kiyama T, Hirahara K, Tanaka N, Kuwabata S, Torimoto T (2008). Single-step synthesis of gold–silver alloy nanoparticles in ionic liquids by a sputter deposition technique. Chem. Commun..

[37] Liu C-H, Chen X-Q, Hu Y-F, Sham T-K, Sun Q-J, Chang J-B, Gao X, Sun X-H, Wang S-D (2013). One-pot environmentally friendly approach toward highly catalytically active bimetal-nanoparticle-graphene hybrids. ACS Appl. Mater. Interfaces.

[38] Li X, Hong X (2016). PdPt@Au core@shell nanoparticles: alloyed-core manipulation of CO electrocatalytic oxidation properties. Catal. Commun..

[39] Tada H, Suzuki F, Ito S, Akita T, Tanaka K, Kawahara T, Kobayashi H (2002). Au-core/Pt-shell bimetallic cluster-loaded TiO_2_. 1. Adsorption of organosulfur compound. J. Phys. Chem. B.

[40] Bus E, van Bokhoven JA (2007). Electronic, geometric structures of supported platinum, gold, and platinum-gold catalysts. J. Phys. Chem. C.

[41] Teng X, Feygenson M, Wang Q, He J, Du W, Frenkel AI, Han W, Aronson M (2009). Electronic and magnetic properties of ultrathin Au/Pt nanowires. Nano Lett..

[42] Zhang P, Sham T (2003). X-ray studies of the structure and electronic behavior of alkanethiolate-capped gold nanoparticles: the interplay of size and surface effects. Phys. Rev. Lett..

[43] Lv J-J, Zheng J-N, Wang Y-Y, Wang A-J, Chen L-L, Feng J-J (2014). A simple one-pot strategy to platinum–palladium@palladium core–shell nanostructures with high electrocatalytic activity. J. Power Sources.

[44] Zhang H-J, Lu L-L, Kawashima K, Okumura M, Haruta M, Toshima N (2015). Synthesis and catalytic activity of crown jewel-structured (IrPd)/Au trimetallic nanoclusters. Adv. Mater..

[45] Xia Y, Wu HB, Li N, Yan Y, Lou XW, Wang X (2015). One-pot synthesis of Pt–Co alloy nanowire assemblies with tunable composition and enhanced electrocatalytic properties. Angew. Chem. Int. Ed..

