# Supplementary material for: Synergistic Effects in CNTs-PdAu/Pt Trimetallic Nanoparticles with High Electrocatalytic Activity and Stability
Source: Nanomicro Lett. 2017 Jul 18;9(4):48. doi: 10.1007/s40820-017-0149-1 (PMC6199041; doi:10.1007/s40820-017-0149-1)
Supplement: Supplementary file 1 — Supplementary material 1 (PDF 689 kb) [file 40820_2017_149_MOESM1_ESM.pdf]

Supporting Information for

Synergistic Effects in CNTs-PdAu/Pt Trimetallic Nanoparticles with High Electrocatalytic Activity and Stability

Xin-Lei Cai[1], Chang-Hai Liu[2,*], Jie Liu[1], Ying Lu[1], Ya-Nan Zhong[1], Kai-Qi Nie[1], Jian-Long Xu[1], Xu Gao[1], Xu-Hui Sun[1], Sui-Dong Wang[1,*]

[1]Institute of Functional Nano & Soft Materials (FUNSOM), Jiangsu Key Laboratory for Carbon-Based Functional Materials & Devices, Soochow University, Suzhou, Jiangsu 215123, People's Republic of China

[2]School of Materials Science & Engineering, Jiangsu Collaborative Innovation Center of Photovoltaic Science and Engineering, Changzhou University, Changzhou, Jiangsu 213164, People's Republic of China

*Corresponding authors. E-mail: wangsd@suda.edu.cn, liuch@cczu.edu.cn

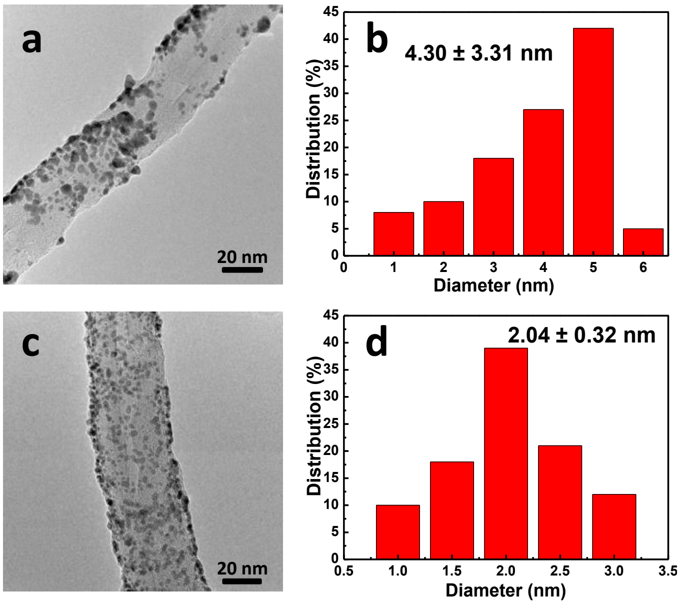

Fig. S1 TEM images and size distribution of: **a** and **b** CNTs-Au/Pt NPs, and **c** and **d** Pd/Pt NPs

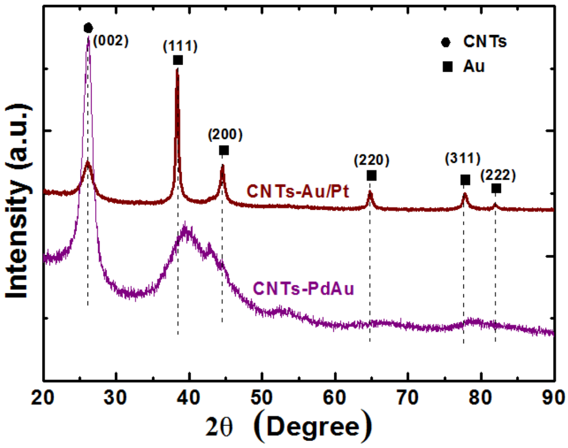

Fig. S2 XRD patterns of CNTs-Au/Pt NPs and Pd/Au NPs

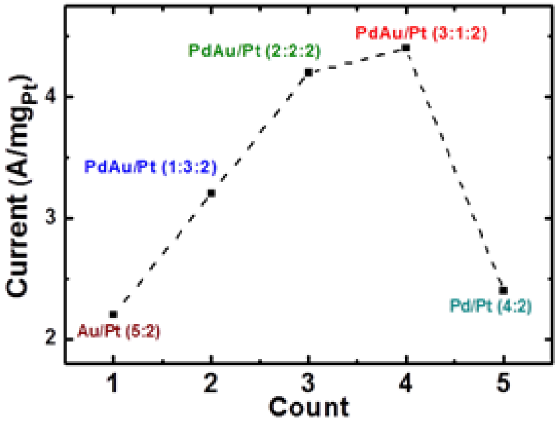

Fig. S3 Volcano plot between current and different kinds of CNTs-supported alloy catalysts
